# Revisiting the Rearrangement of Dewar Thiophenes

**DOI:** 10.3390/molecules25020284

**Published:** 2020-01-10

**Authors:** Sara Gómez, Edison Osorio, Eugenia Dzib, Rafael Islas, Albeiro Restrepo, Gabriel Merino

**Affiliations:** 1 Scuola Normale Superiore, Classe di Scienze, Piazza dei Cavalieri 7, 56126 Pisa, Italy; 2 Facultad de Ciencias Naturales y Matemáticas, Universidad de Ibagué, Carrera 22 Calle 67, Ibagué, Colombia; edison.osorio@unibague.edu.co; 3 Departamento de Física Aplicada, Centro de Investigación y de Estudios Avanzados, Unidad Mérida. Km 6 Antigua Carretera a Progreso, Apdo. Postal 73, Cordemex, 97310 Mérida, Mexico; eugenia.dzib@cinvestav.mx; 4 Departamento de Ciencias Químicas, Facultad de Ciencias Exactas, Universidad Andres Bello, Av. República 275, Santiago, Chile; rafael.islas@unab.cl; 5 Instituto de Química, Universidad de Antioquia UdeA, Calle 70 No. 52-21, Medellín, Antioquia, Colombia

**Keywords:** fluxionality, Dewar thiophenes, reaction mechanisms, one-electron bonds

## Abstract

The mechanism for the walk rearrangement in Dewar thiophenes has been clarified theoretically by studying the evolution of chemical bonds along the intrinsic reaction coordinates. Substituent effects on the overall mechanism are assessed by using combinations of the ring (R = H, CF_3_) and traveling (X = S, S = O, and CH_2_) groups. The origins of fluxionality in the S–oxide of perfluorotetramethyl Dewar thiophene are uncovered in this work. Dewar rearrangements are chemical processes that occur with a high degree of synchronicity. These changes are directly related to the activation energy.

## 1. Introduction

**Scheme 1:**
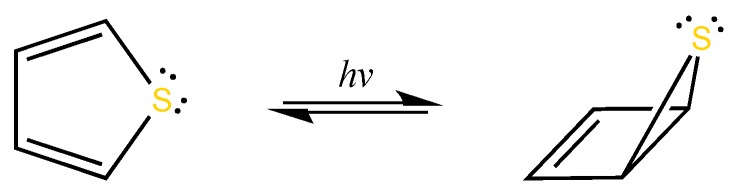
Photoisomerization of thiophene to Dewar thiophene.

Dewar thiophenes are bicyclic structural isomers of thiophenes, where the carbon atoms bonded to the S atom have *sp*^3^ hybridization (Scheme 1). Thiophenes are planar and have 6π-electrons in the five-membered ring, including an S electron pair, so they are classified as aromatic. Conversely, because of the isolated double bond, Dewar thiophenes are non-aromatic. Photoisomerization of the parent thiophene is a standard method to produce Dewar thiophenes, and according to Kobayashi and Kumadaki, in some cases, despite being highly strained, Dewar structures are stable and may be kept in a refrigerator for several years [1].

**Scheme 2:**
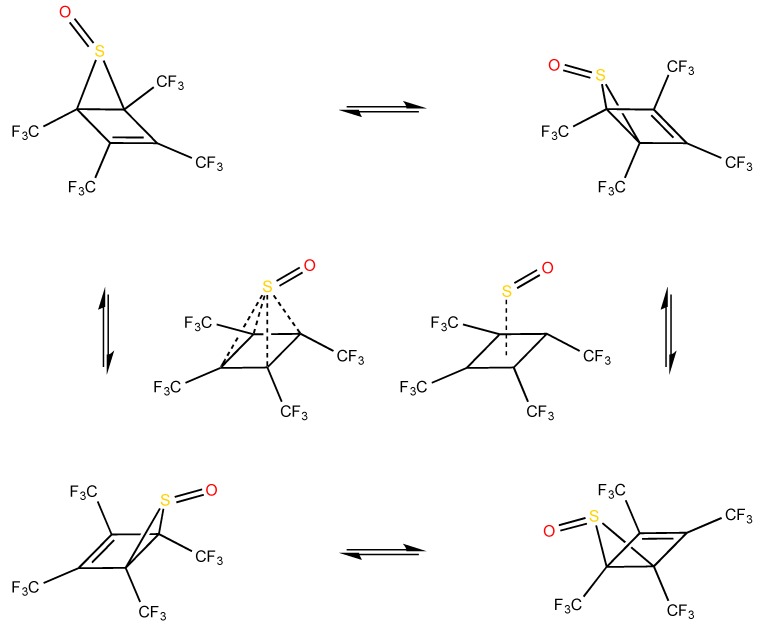
Rearrangement of the S-oxide Dewar perfluorotetramethyl thiophene. The four equivalent structural possibilities [2] may be succinctly represented by either one of the two structures highlighted at the center.

Bushweller and co-workers discovered a remarkable dynamical process for the S-oxide of the Dewar perfluorotetramethyl thiophene in 1976: their ^19^F-NMR spectra show a sharp singlet at −78.5 °C; thus, all fluorine atoms are equivalent. This structural puzzle is consistent with a fast rearrangement of the S = O group and the C = C double bond around all possible equivalent configurations (fluxionality) [2]. At −78.5 °C, each walk rearrangement step must overcome an energy barrier not exceeding 7.0 kcal mol^−1^. In other words, there is full delocalization of the double bond and S = O moiety, as well as planarity of the cyclic carbon core, as is illustrated in the highlighted structures at the center of Scheme 2. Remarkably, the same rearrangements oppose considerably higher barriers for the parent pristine (non-oxidized) perfluorotetramethyl thiophene (~22 kcal mol^−1^ at 157 °C). 

The mechanism for Dewar rearrangements has been a subject of study since its discovery [2,3,4,5,6]. The difficulties encountered in deciphering this transformation were summarized by Schleyer and co-workers, who wrote, “*this low-barrier rearrangement should correspond—at least formally—to a thermal* [1,3] *suprafacial sigmatropic shift, a process forbidden by the Woodward–Hoffmann rules. To overcome this mechanistic conundrum, Lemal et al*. *proposed the participation of a nonbonding sulphur lone pair, implying an alternative nonpericyclic mechanism*” [6]. Some of us have been interested in rearrangements in organic systems [7,8,9,10,11] for a long time and this system represents an exciting case to apply different tools to explain its dynamical behavior. 

**Scheme 3:**
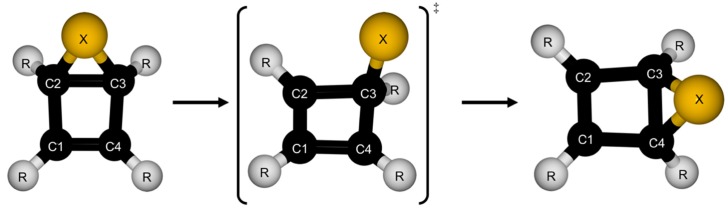
The elemental step of the Dewar rearrangement.

Herein, we attempt to provide a very detailed picture of the intricacies involved in the mechanism of Dewar rearrangements. As test cases, we chose a set of combinations of substituents in the ring (−R) and in the traveling groups (−X), as depicted in Scheme 3 (X = S, S = O, and CH_2_; R = H, CF_3_). In particular, we focus on the evolution of chemical bonds along the intrinsic reaction coordinates (IRC) and derive conclusions regarding synchronicity and the effects of substituents in the overall mechanism. In addition, we study the reaction path under the perspective of the reaction force [12,13,14].

## 2. Computational Details

The lowest energy hypothetical path connecting reactants with products along the reaction coordinate in the potential energy surface is called the intrinsic reaction coordinate [15]. Most reactions encounter a maximum in this path, around which the entire transition state theory has been constructed. One useful tool to dissect the IRC comes from the consideration of the reaction force: [12,13,14,16,17] in conservative systems, the net force acting on the system may be obtained from the derivative of the potential energy:

(1) $$F=-\frac{dV}{d\xi }$$

For every step, five important points are discerned: The reactants, transition state, and products, all are equilibrium points. The activated reactants, ξ_R_^*^, and activated products, ξ_P_^*^, correspond to the minimum and maximum points along the force profile (or equivalently, they correspond to the inflection points in the IRC). In this partition of the reaction profile, the system opposes the progress of the chemical in the region of negative (retarding) forces and drives the chemical reaction in the region of positive forces. Bader’s theory, Natural Bond Orbital (NBO), and Adaptive Natural Density Partitioning (AdNDP) methods will be discussed below when appropriate. Geometry optimizations, vibrational frequencies, and IRCs were carried out using Gaussian 09 [18] at the PBE0 [19,20]/def2-TZVP [21,22] level corrected with Grimme D3 empirical dispersion [23] (PBE0-D3/def2-TZVP). NBO calculations were conducted using the NBO 6.0 suite [24]. AdNDP calculations were carried out using the newly released alpha.1.0 version [25,26].

## 3. Structures

We located the transition states connecting the Dewar thiophenes depicted in Scheme 3. Relevant geometrical parameters for the specific case of perfluorotetramethyl thiophene (X = S=O and R = CF_3_) are listed in Table 1. The following inventory of bond breaking/formation is established: while C2-S9 is breaking and C4-S9 is forming, C3-S9 remains unaltered. Thus, while C2 changes from *sp*^3^ to *sp*^2^ character, C4 switches from *sp*^2^ to *sp*^3^, and C3 remains *sp*^3^ along the step. Bond lengths rearrange accordingly. Visual inspection suggests that in the transition state, the S atom is connected only to C3. 

Our computations show that for the minima, there are at least two types of F atoms that cannot be told apart by the experiment because of the fast nature of the rearrangement (*vide supra*). The ^19^F NMR spectrum of this molecule at −78.5 °C shows a sharp singlet resonance, indicating a fast rearrangement. It suggests an “average” structure (at the center of Scheme 2), where the traveling group is delocalized above the ring. We located this structure, but it is a high order saddle-point connecting the transition states for the Dewar rearrangement. 

## 4. Reaction Profiles and Forces

Our DFT computations accurately reproduce the experimental activation energies (at −137.8 and 157 °C). Actually, DLPNO–CCSD (T) energies clearly show (see Table S1 and the Supplementary Materials for details) that accurate treatment of electron correlation makes no difference in energy barriers. Thus, our selected methodology is strongly supported. Activation energies for Dewar rearrangements are sensibly affected by the nature of the traveling group on top of the ring (Table 2). Conversely, the identity of the ring substituents does not seem to affect the rearrangement. Since substituting the traveling group by −CH_2_ still leads to the same Dewar rearrangement, with activation energies somewhat larger than for the -S case, serious doubts are cast on the mechanism suggested by Bushweller and co-workers [2], which invokes the active role of an electron pair in the traveling group (−CH_2_ has none). Furthermore, our results suggest that should there be Dewar rearrangement, the reaction mechanism is unaltered in the context of the primitive changes that drive the chemical transformation, the only differences occurring in the magnitude of activation energies. Thus, the nature and geometry of the transition state are independent of the identity of both X and R.

**Figure 1:**
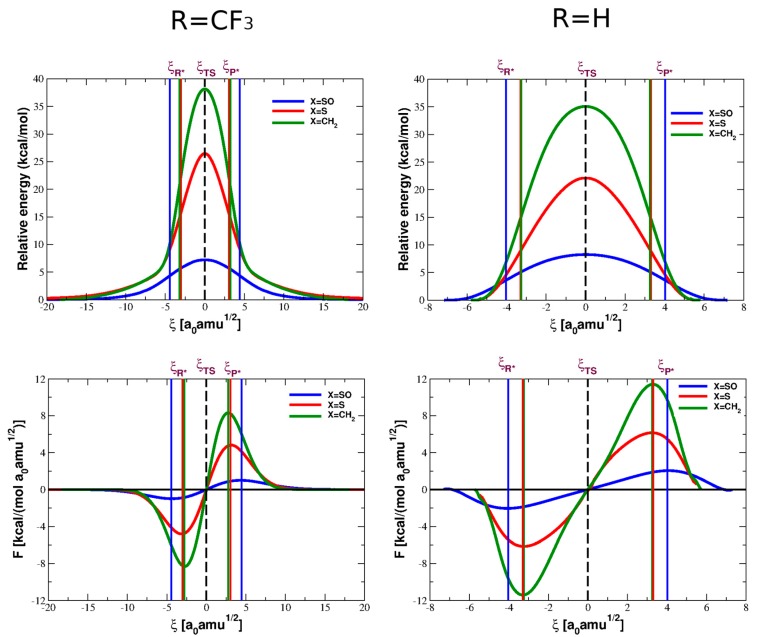
Energy profiles (top) and the corresponding reaction forces (bottom) as given by Equation (1) for all Dewar rearrangements in this work. All computations at the PBE0-D3/def2-TZVP level.

**Figure 2:**
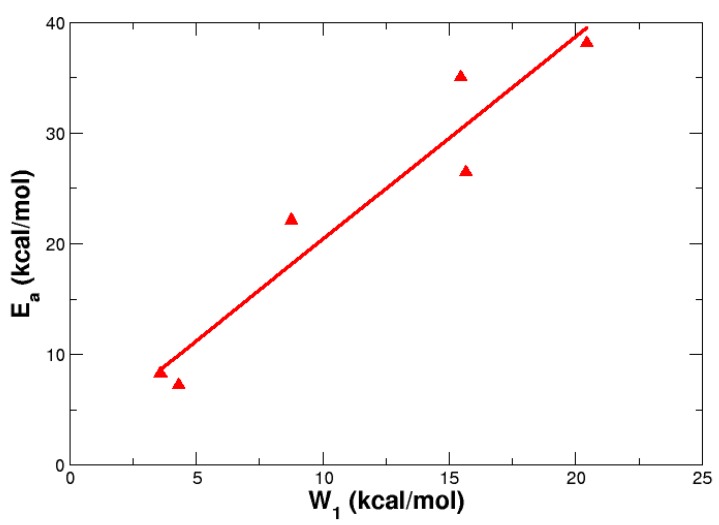
Correlation between activation energies and W_1_, the work needed to take the reacting system to the coordinate of the activated reactants: $W_{1}\left(\xi_{R}\to \xi_{R*}\right)=-\int_{\xi_{R}*}^{\xi_{R}}F\left(\xi \right)d\xi$. Linear regression equation: E_a_ = 2.0637 + 1.8327 W_1_, R^2^ = 0.923.

A general description of the chemical reaction as derived from the reaction force is quite illustrative (see Figure 1). For all cases, up to ξ_TS_, the reacting system experiences a negative (retarding) force that opposes the reaction. Once the TS is overcome, going downhill, a driving net force takes the reacting system first to the activated products and then to the final products. However, this force is not constant; indeed, the opposition to the chemical reactions steadily increases from ξ_R_, the coordinates of the reactants to ξ_R*_, the coordinates of the activated reactants, then, this opposing force steadily diminishes until it vanishes at the transition state. Some authors equate the region of increasing opposition to mainly structural changes, needed to take the reactants from their starting geometries to the proper configurations required so that the electronic activity responsible for the bond breaking/formation may take place [17,27]. As expected, larger retarding forces lead to larger activation energies. This is not just a simple trend, the work involved in going from reactants to activated reactants (W_1_) is correlated (Figure 2) with the activation energies, as has been reported for other reactions [28].

## 5. Evolution of Bonding

### 5.1. Bond Orders and Their Derivatives

**Figure 3:**
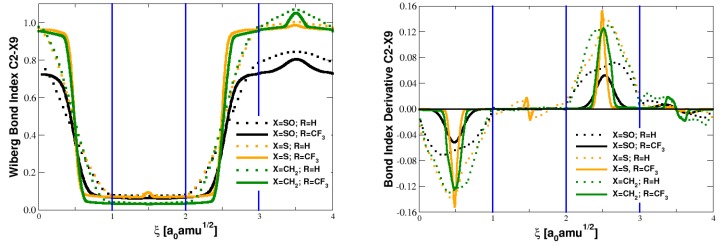
Evolution of the C2-X9 bond along an entire cycle (four steps in total, separated by vertical blue solid lines) of Dewar rearrangements as depicted in Scheme 3.

Exciting observations are drawn from Figure 3, which follows the evolution of the C2-X9 bond order and its derivative along the entire cycle of Dewar rearrangements depicted in Scheme 3 for all substituents. Interestingly, the C-S bonds that change during the rearrangement are already somewhat weaker than traditional C-S bonds (Wiberg bond indices of 1.04 in CH_3_-SH, 0.95 in (CH_3_)_2_S = O, and 1.03 (CH_3_)_2_S). Thus, in both R = −CF_3_ and R = −H cases, the C2-S9 is weakened by the combined action of the strain on the C-S bonds induced because of the three-membered cycle on one the hand, and because of the presence of the oxygen atom, on the other. Indeed, as seen in the left panel of Figure 3, the bond order for the C2-S9 bond is consistently lower along the four consecutive Dewar rearrangements than the bond orders of the corresponding C2-S9 (bare S, no oxide) and C2-C9 (from CH_2_) regardless of the nature of the substituents at the ring. Again, this rationalization of the reaction mechanism also opposes the original view of Bushweller and co-workers, who argue in favor of an active role of a lone pair in the sulfur atom [2]. There are no available lone pairs in the −CH_2_ group. However, the corresponding Dewar thiophene still undergoes the rearrangement with a barrier similar in magnitude to that of the bare X = S group, which has two lone pairs but leads to non-fluxional rearrangements. The derivative of the C2-S9 bond order (right panel, Figure 3) along the four Dewar rearrangements also reveals important mechanistic details. First, the nature of the bond breaking/formation is evident from the sign of the derivative (which is also evident from the decreasing/increasing regions of the bond order plots). Second, clearly for the fluxional cases (both S-oxides), electron activity, that is, C2-S9 bond breaking/formation starts very early in the reaction relative to the other cases. Finally, given the cyclic character of the Dewar rearrangement, for any single step, it is evident from the left panel in Figure 3 that fluxionality is directly tied to the strength of the C-S bond that acts as a pivot for the transfer of the S = O group: The bond order for this C2-S9 bond is sensibly smaller than for the non-fluxional cases.

### 5.2. Evolution of the Dewar Rearrangement in S-oxide Perfluorotetramethyl Thiophene

**Figure 4:**
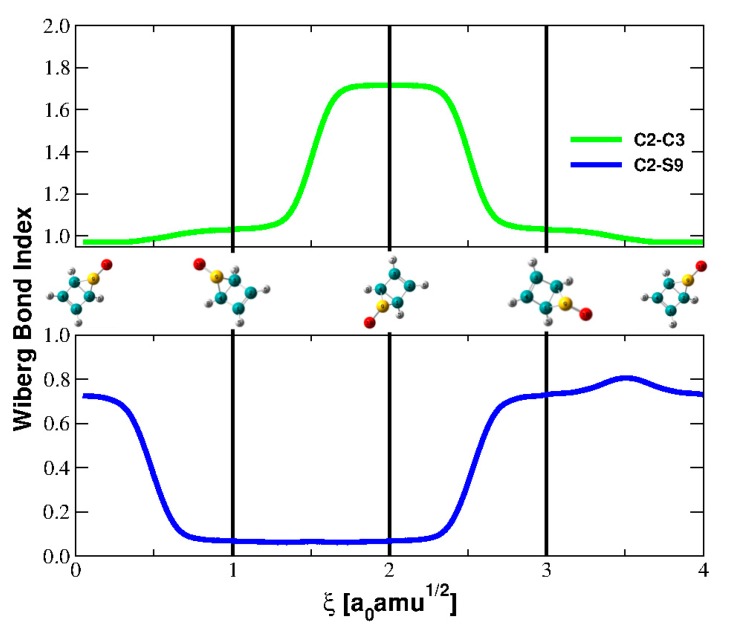
Evolution of the C2-C3 and C2-S9 bonds along an entire cycle of Dewar rearrangements, as depicted in Scheme 3 for S-oxide perfluorotetramethylthiophene.

Since the qualitative features of the mechanism of Dewar rearrangements are independent of the identity of the X and R groups, we now turn our attention to the systems for which fluxionality has been experimentally established even at low temperatures, that is, X = S = O and R = CF_3_. From the perspective of primitive changes, due to the symmetry of all C-C and C-S bonds, we arbitrarily chose the C2-C3 and C2-S9 bonds in Figure 4 to follow their evolution. For the majority of the four elemental steps (about 75% of the reaction path), any given C-C bond may be described as a single bond, while only for one short lapse (about 25% of the reaction path) it may be considered a double bond. Conversely, for about 50% of the entire reaction path covering four rearrangements, any given C-S bond is present, either as a pivot for the transfer of the S = O group or as a formal bond in a three-membered C-S-C cycle.

**Figure 5:**
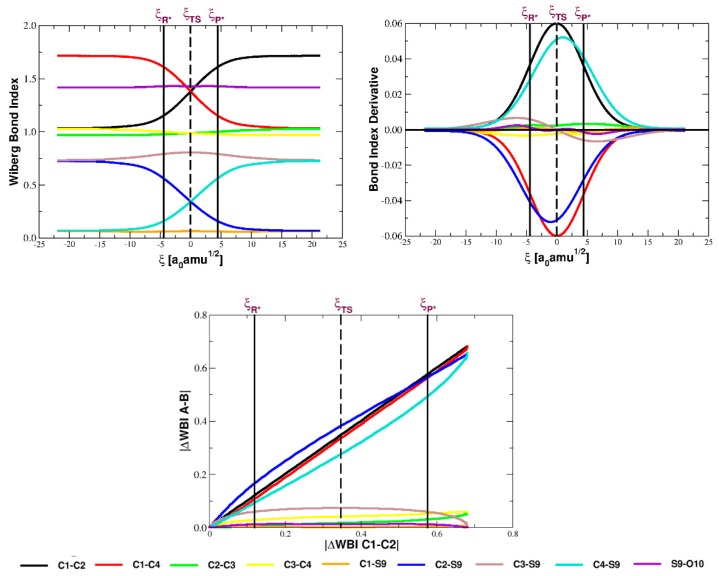
Evolution of all relevant bonds during one walk step of the Dewar rearrangement in S-oxide perfluorotetramethylthiophene. Bond orders along the reaction coordinate are pictured in the top left panel, their derivatives in the top right panel, and the changes relative to the chosen C1-C2 reference bond are plotted at the bottom. See Figures S1–S3 for the other studied Dewar thiophenes.

Recall the inventory of bond breaking/formation stated above: Out of the nine bonds relevant for the rearrangement of the S-oxide perfluorotetramethylthiophene, for a single step (Scheme 3), a total of four bonds are significantly changed (C1-C4, C1-C2, C2-S9, C4-S9), while one (C3-S9) bond serves as a lever for the transfer of the S = O group. Thus, during the Dewar rearrangement in Scheme 3, the C1-C4 double bond is transformed into a single bond, the single C1-C2 bond is transformed into a double bond, C2-S9 disappears, and C4-S9 is formed. Figure 5 shows the evolution of these bonds during one walk step. The active participation of two C-C bonds and two C-S bonds is evident, while the pivotal nature of one of the C-S bonds is reflected in only small variations of the bond order during the transition state (we already related this bond order to the corresponding activation energy), the other four bonds may be considered spectators. Note that from the perspective of primitive changes, our results indicate that Dewar rearrangements are chemical processes that occur with a high degree of synchronicity: bonds sharing a common atom evolve at the same rate, thus, on the one hand, the single C1-C2 bond is converted to a double bond at the same rate as the C1-C4 double bond is transformed into a single bond; while on the other hand, the C2-S9 bond disappears at the same rate as the C4-S9 bond is being formed. This simultaneous evolution of chemical bonding may be better appreciated by a criteria for synchronicity developed elsewhere [28] and plotted at the bottom panel of Figure 5: If the C1-C2 bond is taken as reference, the other bonds involved in the chemical transformation depart from the initial bond order at approximately the same rate as this bond, while the bonds not taking part in the rearrangement do not change. While all the primitive changes are occurring, the pivotal C3-S9 bond changes only slightly, however, the evolution of this bond is quite important because changes in this bond are directly related to the activation energy in the fluxional case (*vide supra*). 

### 5.3. Analysis of the Electron Densities

Bader theory [29,30,31,32,33,34] provides useful tools to analyze bonding and electron delocalization [35] once the molecular problem has been solved. The bicyclic nature of the Dewar thiophene is well-established by the presence of two ring critical points. In the same line, transition states (Scheme 3) are non-bicyclic; thus, the pivoting nature of one of the C-S bonds is also established.

**Figure 6:**
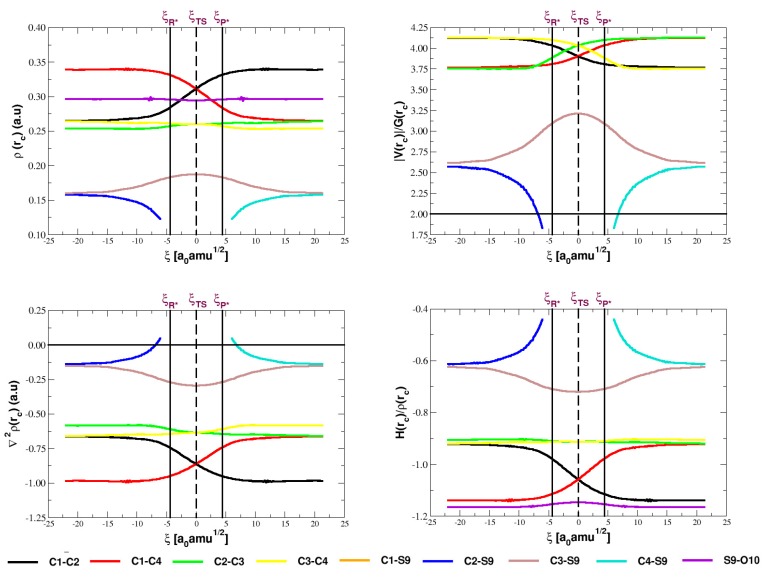
Electron density descriptors of the evolution of the nature of bonding interactions in the Dewar rearrangement in S-oxide perfluorotetramethylthiophene (Scheme 3). See Figures S4–S7 for the other studied Dewar thiophenes.

In this work, we gather insight into the nature of bonding interactions and the evolution of bonding using descriptors calculated at the bond critical points (BCPs). In particular, we are interested in the following four descriptors: (i) the amount of electron density at the BCPs (top left panel, Figure 6), which is directly related to the nature of a particular bonding interaction in the sense that larger accumulations of electron densities in the internuclear regions are (intuitively) characteristic of covalent bonds, while charge depletion on those regions describes closed-shell (long-range, ionic, etc.) interactions. (ii) Local application of the virial theorem by Espinosa and co-workers [36] lead them to suggest that the ratio of potential to kinetic energy (top right panel, Figure 6) is indicative of the nature of bonding interactions: while ratios in the [0,1] interval describe long-range interactions, ratios larger than 2 describe covalent interactions, and ratios in the [1,2] interval are classified as being of intermediate character, with covalent and closed-shell contributions. (iii) The Laplacian of the electron density (bottom left panel, Figure 6) indicates local concentration/depletion of charge [31], while its sign calculated at some point describes local curvature of the electron distribution. (iv) The sign of the total energy density (bottom right panel, Figure 6) is also a good qualitative descriptor of the nature of bonding interactions: the total energy is the sum of the potential (always negative, stabilizing) and kinetic (always positive, destabilizing) energies. Thus, on the one hand, BCPs for which the total energy is positive, represent a situation where the local kinetic energy is larger than the local potential energy, disfavoring local concentration of charge, again, proper of long-range or ionic interactions; on the other hand, by the same token, BCPs for which the total energy is negative, characterize points of local concentration of charge, characteristic of covalent bonding.

Fittingly, when analyzing electron density-based descriptors, the plots in Figure 6 provide a picture entirely consistent with the above bond order-based description of the evolution of chemical bonds during the Dewar rearrangement of S-oxide perfluorotetramethylthiophene that may be summarized as follows: (i) There is an expected increase (decrease) in the electron density at the BCPs associated to the chemical bonds in the process of being formed (broken) (ii) Single C-C bonds common to both cycles (C2-C3 in the particular step shown in Scheme 3) are weaker than other single C-C bonds (iii) For all formal bonds, V/G, the Laplacian of the electron density, and the total energy density are consistent in describing covalent interactions (iv) All descriptors predict a strengthening of the pivoting C3-S9 bond that maximizes right at the transition state (v) According to these criteria, the formation of the C4-S9 bond seems to lag behind the breaking of the C2-S9 bond, thus apparently opposing the highly synchronic view of the process suggested by the above analysis of bond orders. A close look at the formalism fixes this apparent inconsistency: a bond order can always be calculated for any given pair of atoms, including the cases where there are no bonding paths. In our case, bond orders for the emerging C4-S9 interaction are very close to zero in the early stages of the rearrangement, where no critical point is found. Similarly, bond orders for the breaking C2-S9 bond are very close to zero in the late stages of the process. 

### 5.4. NBO and AdNDP Analysis

**Figure 7:**
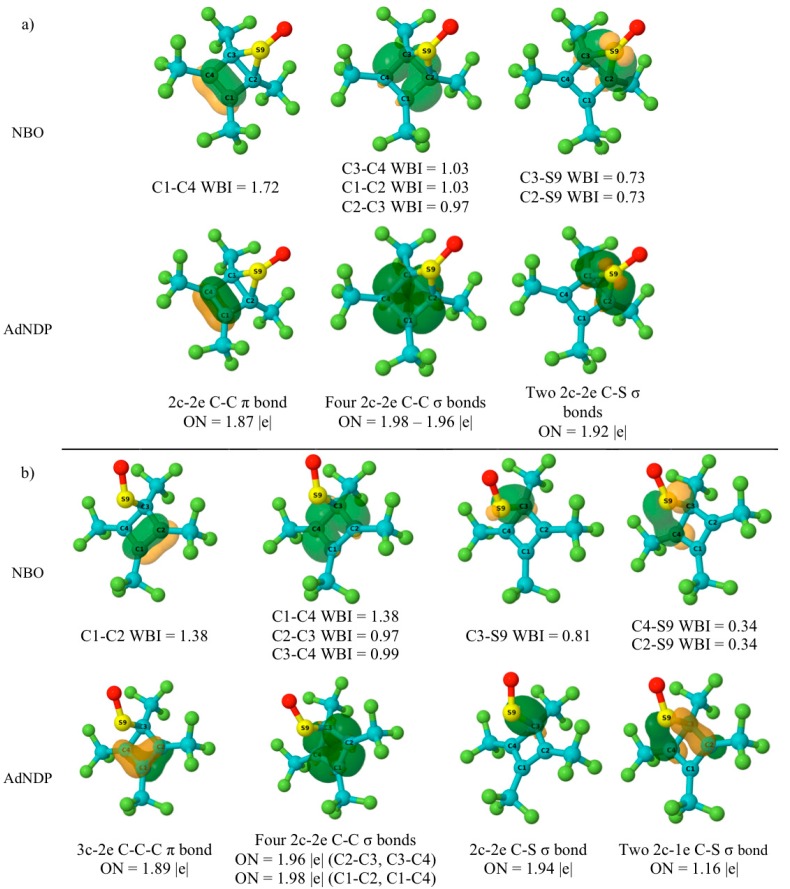
NBO and AdNDP descriptors of chemical bonding in the equilibrium structure (**a**) and for the transition state (**b**) of the fluxional S-oxide perfluorotetramethyl Dewar thiophene. WBI, Wiberg bond indices, and ON, occupancy numbers for each orbital are included.

The theory and principles behind NBO and AdNDP are well-documented elsewhere [25,26,37]. In short, NBO produces a chemically intuitive picture of the electronic structure of molecules in terms of lone pairs and Lewis-type molecular orbitals with double occupancies, localized between pairs of atoms. The adaptive natural density partition (AdDNP) method generalizes this view to account for multicenter bonds sharing two electrons. A very interesting picture of the evolution of bonding during Dewar rearrangements, fully consistent and complementary to the above discussion derived from electron density descriptors, emerges when using localized Lewis-type orbitals and multicenter orbitals. The relevant orbitals and information are summarized in Figure 7 for the fluxional S-oxide perfluorotetramethyl Dewar thiophene case, analogous results for other systems studied here are available in the Supplementary Materials (Table S2). 

For the minima (Figure 7a), both NBO and AdNDP recover the bond inventory established above: one double (C1-C4) bond, three single (C1-C2, C2-C3, C3-C4) bonds, and two additional single (C2-S9, C3-S9) bonds. The WBIs [38] and the occupancy number suggest, as expected, that the C2-C3 bond is slightly different from the adjacent C1-C2 and C3-C4 bonds. In addition to both C-S bonds having low WBIs, they have comparatively lower electron populations; thus, they appear to be prone to undergo chemical transformations. 

The electronic structure of the transition states (Figure 7b) is quite interesting. Since NBO optimizes orbital occupancies to reproduce the insightful Lewis structures based on lone pairs and 2c-2e bonding orbitals, it is not surprising that according to this method, the double C1-C4 bond has already been transformed into a single bond and that C1-C2 has been transformed into a double bond, as early as in the transition state, and that two other single C-C bonds in the ring are obtained at this stage. Fittingly, the AdNDP method recovers the delocalization of the 2π electrons among three carbon atoms not attached to S at the transition state in a manner that is consistent with both bonds having a WBI of 1.38. The interpretation of the C-S bonds is more challenging. Because of the need to produce doubly occupied 2c-2e orbitals, besides the pivoting C3-S9 bond, NBO assigns two electrons to the bonding σ-orbital emerging because of the C4-S9 bond being formed and no electrons or bonding orbital to the C2-S9 being broken. This is inconsistent with the discussion based on the evolution of bond orders and their derivatives, where it was established that not only C4-S9 and C2-S9 evolve simultaneously, but that they also have the same bond order at the transition state (0.34). The conflict is resolved by asking for a directed multicenter bond in NBO, which nicely reproduces the 3–center C2-S9-C4 bond sharing a total of 1.72 electrons.

The AdNDP method provides an equally inconsistent picture in which two electrons are shared by three (C2-S9-C4) atoms in two σ-bonding orbitals, leading to a sort of partial bonding in both C4-S9 and C2-S9 just as NBO describes. Indeed, according to AdNDP there are 1.71 electrons in this 3c-2e orbital, that is, 0.85 electrons per σ-orbital. We interpret the combination of WBI, NBO, and AdNDP results in the following way: at the transition state for the fluxional rearrangement of S-oxide perfluorotetramethyl thiophene, there are two 2-center bonds, each with a population of 0.85 (≈1) electrons!

To clarify the picture of the one-electron bonds in the C2-S9-C4 reactive center, we conducted a search of 2c–1e bonds in AdNDP and nicely recovered occupancies for the C4-S9 and C2-S9 σ bonds of 1.16 |e|, in total agreement with the just discussed interpretation of WBI, AIM, and NBO results. The corresponding values are listed in Table 3. 2c-1e bonds are not exclusive of the transition state for the fluxional rearrangement of S-oxide perfluorotetramethyl Dewar thiophene. Table 3 shows that these bonds are present in all cases studied here. We notice that besides the above-mentioned link between the weakening of the C3-S9 pivoting bond at the TS, there is a second factor tied to fluxionality: comparatively low populations of the 2c-1e bonds.

## 6. Summary and Conclusions

The origins of the fluxionality discovered by Bushweller and co-workers in 1976 for the rearrangement of the S–oxide perfluorotetramethyl Dewar thiophene are uncovered in this work: the traveling –S = O group faces small barriers to hop among neighboring carbon atoms because of a strengthening of the pivoting C–S bond and because of the formation of two exotic 2–center 1–electron C–S bonds adjacent to the pivot. We extended our study to other Dewar thiophenes and found that activation energies for the corresponding rearrangements are sensibly affected by the nature of the traveling group (–X = –S, –S = O, –CH_2_), while the identity of the ring substituents (–R = –H, –CF_3_) does not seem to affect the energetics of the process. In the same line, the nature and geometry of the corresponding transition states appear independent of the identity of both –X, and –R. Dewar rearrangements are chemical processes that occur with a high degree of synchronicity: the chemical bonds having a common atom evolve at the same rate, converting a single to a double bond, and returning to a single bond. There is consistency in the general description of the mechanism by all descriptors (electron density-, NBO-, AdNDP) used to study the evolution of chemical bonding during the course of Dewar rearrangement of S–oxide perfluorotetramethyl thiophene.

## Figures and Tables

**Table 1:** Relevant geometrical parameters for the S-oxide perfluorotetramethyl thiophene and for the transition state involved in its Dewar rearrangement (see Scheme 3). All distances in Å and all dihedral angles in degrees. *dr* describes the planarity of the ring of carbon atoms while other dihedrals define the deviation of the −CF_3_ groups from the ring. See the Supplementary Materials for the Cartesian coordinates of the remaining cases studied in this work.

Parameter	Reactant	TS	Product
**Bond Distance**			
C1-C2	1.48	1.40	1.35
C1-C4	1.35	1.40	1.48
C2-C3	1.50	1.49	1.49
C3-C4	1.49	1.49	1.5
C1-S9	2.62	2.69	2.62
C2-S9	1.89	2.31	2.61
C3-S9	1.88	1.81	1.88
C4-S9	2.61	2.31	1.89
S9-O10	1.45	1.45	1.45
**Dihedral Angles**			
*dr*	0	0	0
F3C-C1-C2-C3	−170	−171	−169
F3C-C2-C3-C4	143	179	−169
F3C-C3-C4-C1	142	136	140
F3C-C4-C1-C2	−168	179	144

**Table 2:** Activation energies and selected works along the IRC for all reactions studied herein. All energies in kcal mol^−1^. The two known experimental barriers (see [2]) are included inside parentheses.

X	R		E_a_		W_1_	W_2_
		Electronic	Gibbs at 157 °C	Gibbs at −135.8 °C		
S = O	H	8.26	8.25	7.79	3.58	4.68
	CF_3_	7.23	7.57	6.86	4.29	2.94
				(6.7 ± 0.1)		
S	H^a^	22.14	21.16	21.24	8.76	13.38
	CF_3_	26.46	25.06	25.35	15.64	10.82
			(22.1 ± 0.1)			
CH_2_	H	35.06	32.98	33.16	15.46	19.6
	CF_3_	38.13	35.73	36.4	20.42	17.71

^a^ This reaction was also studied by Dorogan and co-workers at the MP2 (fc)/6-311+G (d,p) and B3LYP/6-311+G (d,p) levels of theory [3].

**Table 3:** AdNDP derived orbital occupancies of 2c-1e bonds in the transition states for all Dewar rearrangements studied in this work. There are marginal contributions from C3 for the R = −CH_2_.

	R = CF_3_		R = H	
	Atoms	ON	Atoms	ON
X = SO	C2-S9	1.16	C2-S9	1.21
	C4-S9	1.16	C4-S9	1.21
X = S	C2-S9	1.58	C2-S9	1.66
	C4-S9	1.58	C4-S9	1.66
X = CH_2_	C2-S9	1.37	C2-S9	1.48
	C4-S9	1.37	C4-S9	1.48

## Supplementary Materials

The following are available online, Table S1: Activation energies at different levels of theory, Figure S1: Wiberg bond indices along the reaction coordinate for one particular step of the Dewar rearrangement, Figure S2: Bond index derivatives along the reaction coordinate for one particular step of the Dewar rearrangement, Figure S3: Absolute values of the change in the WBIs with respect to the reactants (∆WBI) for one particular step of the Dewar rearrangement when the C1–C2 bond is taken as a reference, Figure S4: Electron densities in bond critical points along the IRC for one particular step of the Dewar rearrangement, Figure S5: Laplacian of the electron densities in bond critical points along the IRC for one particular step of the Dewar rearrangement, Figure S6: Espinosa’s criterion in bond critical points along the IRC for one particular step of the Dewar rearrangement, Figure S7: Bond parameter criterion in bond critical points along the IRC for one particular step of the Dewar rearrangement, Table S2: AdNDP Results for all TSs. Cartesian coordinates for all transition states and all minima at the PBE0-D3/def2-TZVP level.
