# Supplementary material for: Revisiting the Rearrangement of Dewar Thiophenes
Source: Molecules. 2020 Jan 10;25(2):284. doi: 10.3390/molecules25020284 (PMC7024393; doi:10.3390/molecules25020284)
Supplement: Supplementary file 1 [file molecules-25-00284-s001.pdf]

Supplementary Material

Revisiting the Rearrangement of Dewar Thiophenes

Sara Gómez[1], Edison Osorio[2], Eugenia Dzib[3], Rafael Islas[4], Albeiro Restrepo[5,*] and Gabriel Merino[3,*]

[1]Scuola Normale Superiore, Classe di Scienze, Piazza dei Cavalieri 7, 56126, Pisa, Italy
[2]Facultad de Ciencias Naturales y Matemáticas, Universidad de Ibagué, Carrera 22 calle 67, Ibagué, Colombia
[3]Departamento de Física Aplicada, Centro de Investigación y de Estudios Avanzados, Unidad Mérida. Km 6 Antigua Carretera a Progreso. Apdo. Postal 73, Cordemex, 97310, Mérida, Yuc., México
[4]Departamento de Ciencias Químicas, Facultad de Ciencias Exactas, Universidad Andrés Bello, Av. República 275, Santiago, Chile
[5]Instituto de Química, Universidad de Antioquia UdeA, Calle 70 No. 52-21, Medellín, Colombia

*Correspondence: gmerino@cinvestav.mx; albeiro.restrepo@udea.edu.co

Contents

1 Activation energies at different levels of theory **S2**

2 Descriptors of the evolution of bonding for all reactions studied here **S3**
2.1 Bond Indices, Bond Index Derivatives and Synchronicity S3
2.2 Electron density Analysis S6
2.3 AdNDP Results for all TSs S10

3 Optimized geometries at the PBE0-D3/def2-TZVP level **S11**
3.1 Cartesian coordinates for all transition states S11
3.2 Cartesian coordinates for all minima S14

1 Activation energies at different levels of theory

Table S1: Activation energies for all reactions studied in the manuscript. All energies in kcal mol^{-1}. All electronic energies corrected for the PBE0–D3 ZPE. The two known experimental barriers (*J. Am. Chem. Soc.* 1977, 99, 629–631) are included inside parentheses. All calculations using the def2–TZVP the basis set.

X	R	Electronic		Gibbs	
		PBE0–D3	DLPNO–CCSD(T)	PBE0–D3	PBE0–D3
				T = 157 °C	T = -135.8 °C
S=O	H	8.26	7.52	8.25	7.79
	CF_3	7.23	6.28	7.57	6.86
					(6.7 ± 0.1)
S	H	22.14	20.91	21.16	21.24
	CF_3	26.46	24.45	25.06	25.35
				(22.1 ± 0.1)	
CH_2	H	35.06	32.31	32.98	33.16
	CF_3	38.13	35.50	35.73	36.40

2 Descriptors of the evolution of bonding for all reactions studied here

2.1 Bond Indices, Bond Index Derivatives and Synchronicity

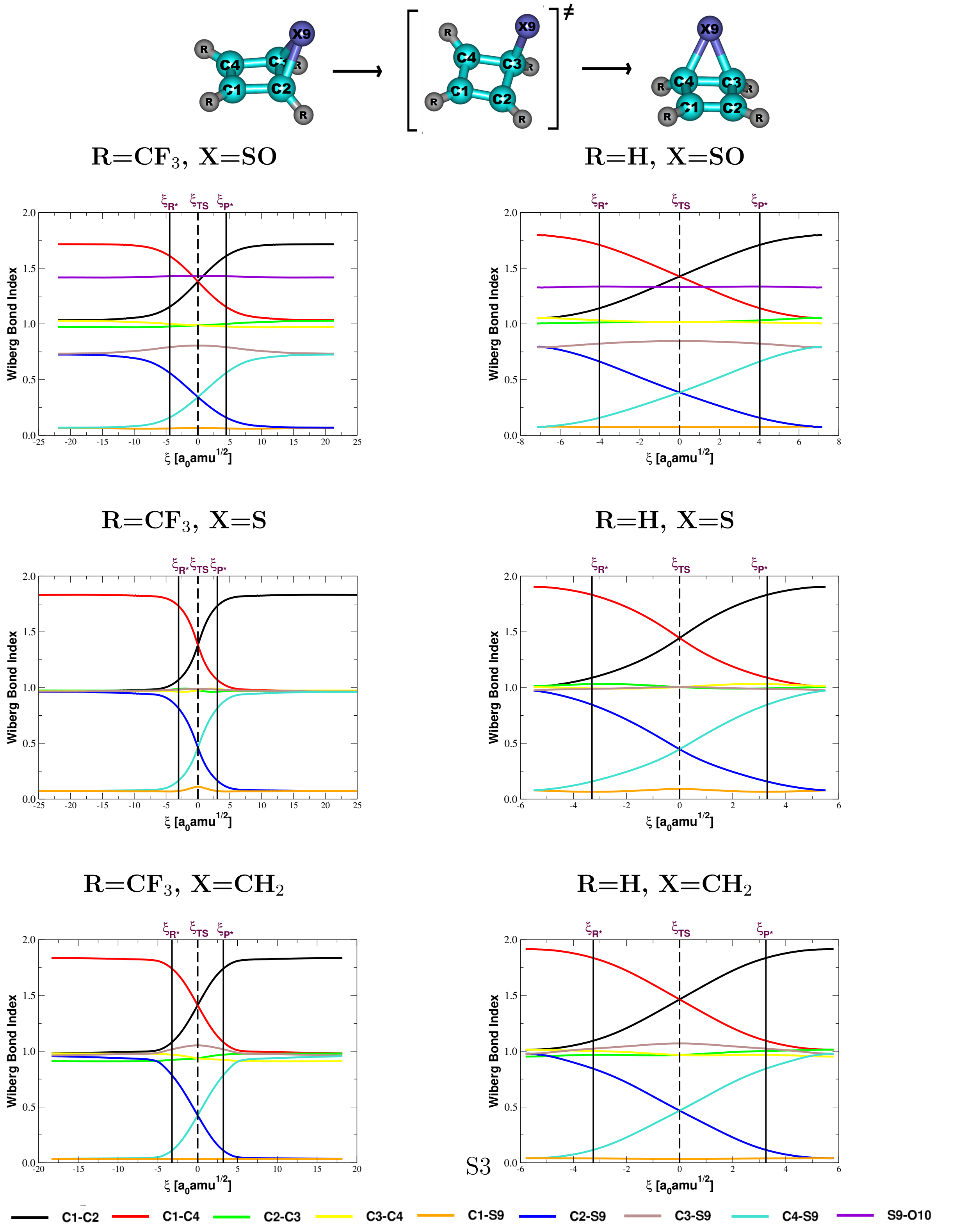

Figure S1: Bond orders along the reaction coordinate for one particular step of the Dewar rearrangement.

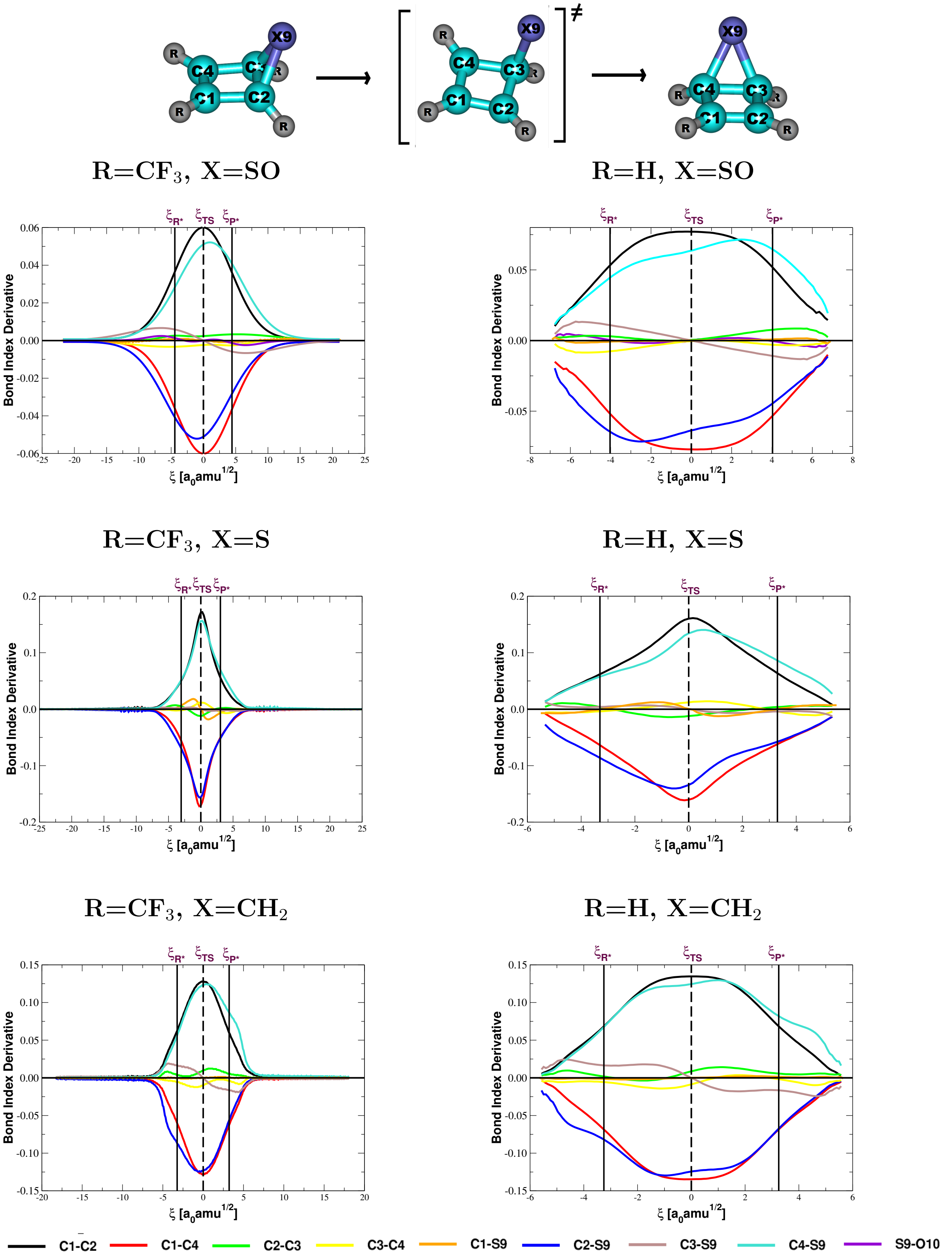

Figure S2: Bond order derivatives along the reaction coordinate for one particular step of the Dewar rearrangement.

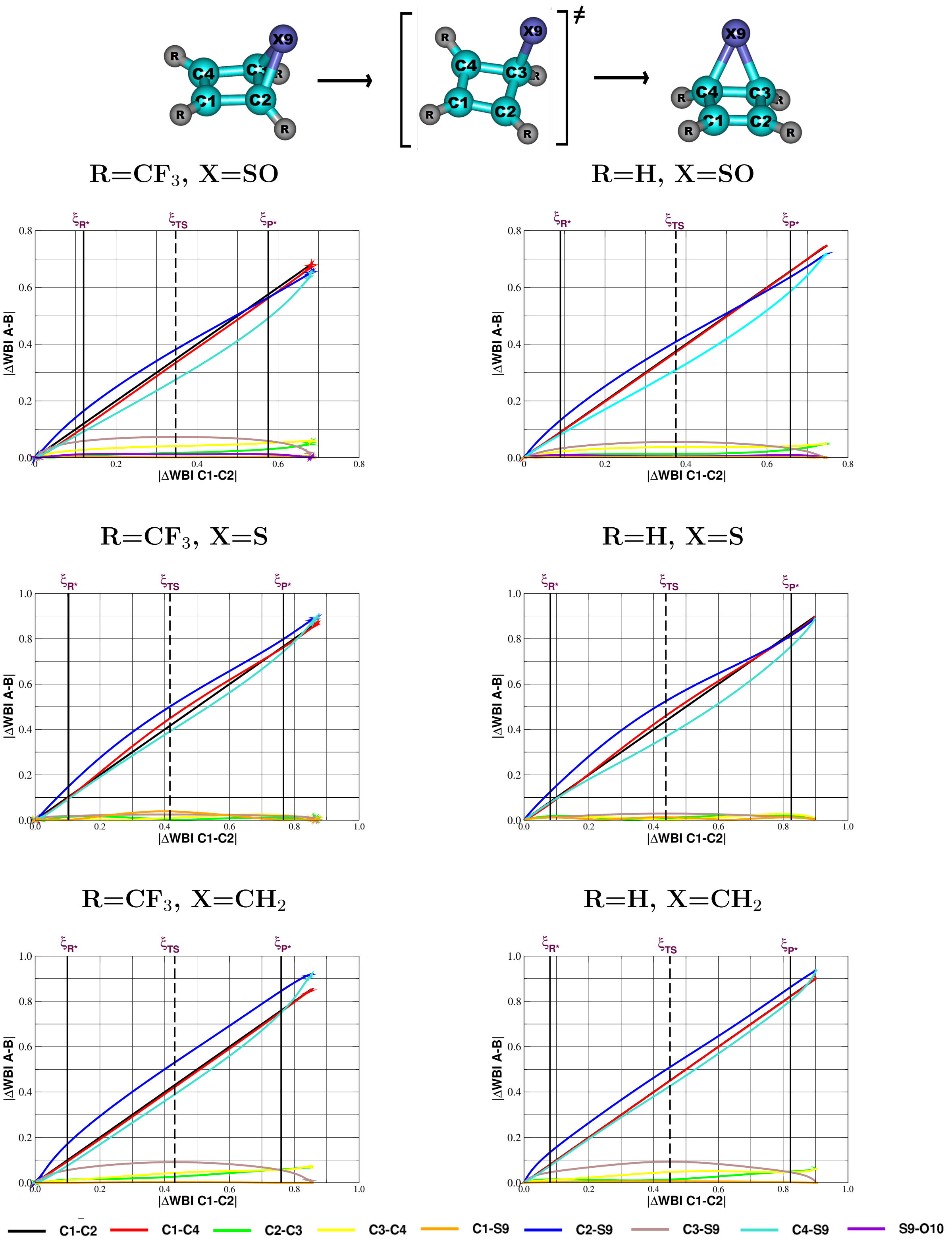

Figure S3: Absolute values of the change in the WBIs with respect to the reactants (ΔWBI) for one particular step of the Dewar rearrangement when the C1–C2 bond is taken as a reference.

2.2 Electron density Analysis

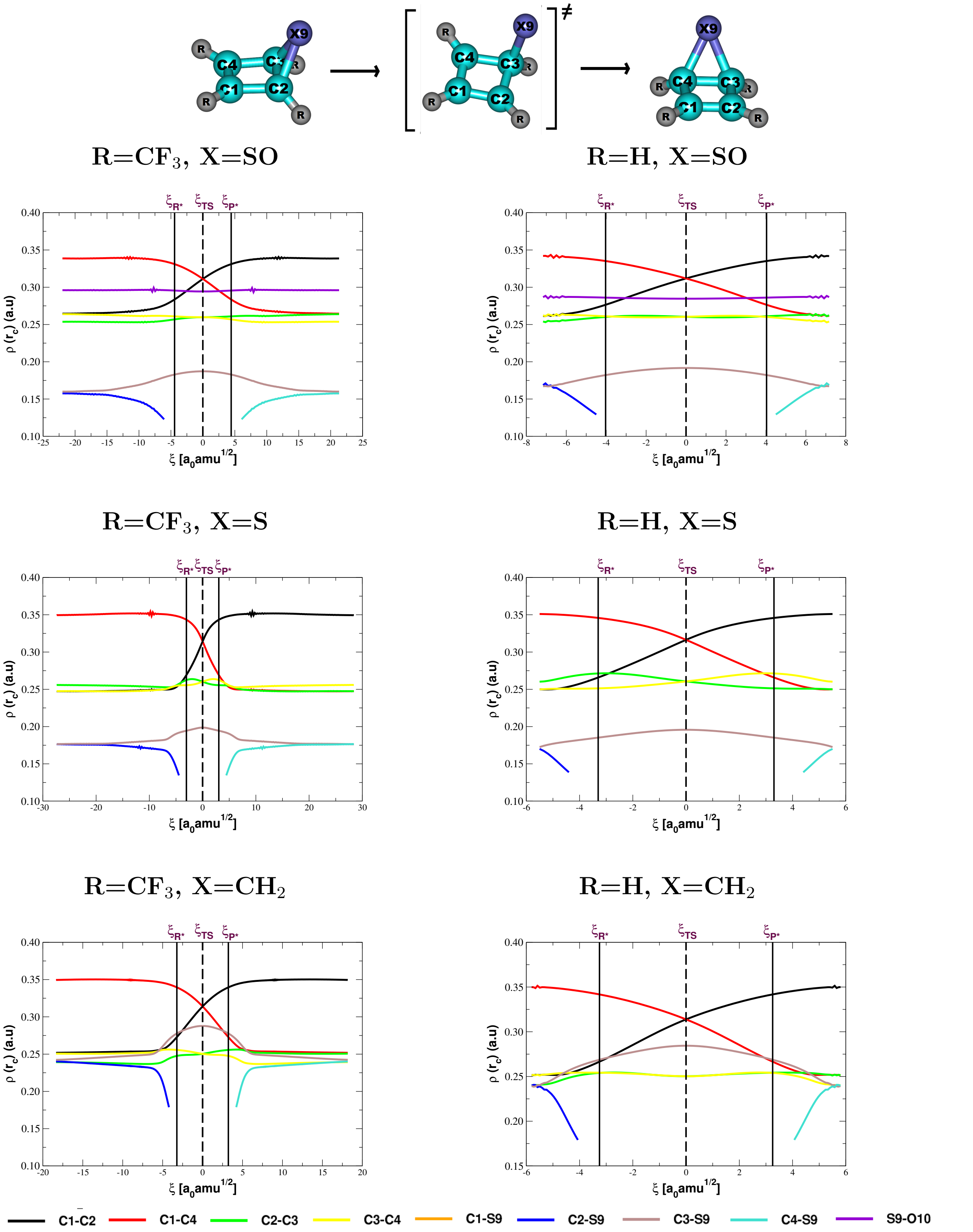

Figure S4: Electron densities in bond critical points along the IRC for one particular step of the Dewar rearrangement.

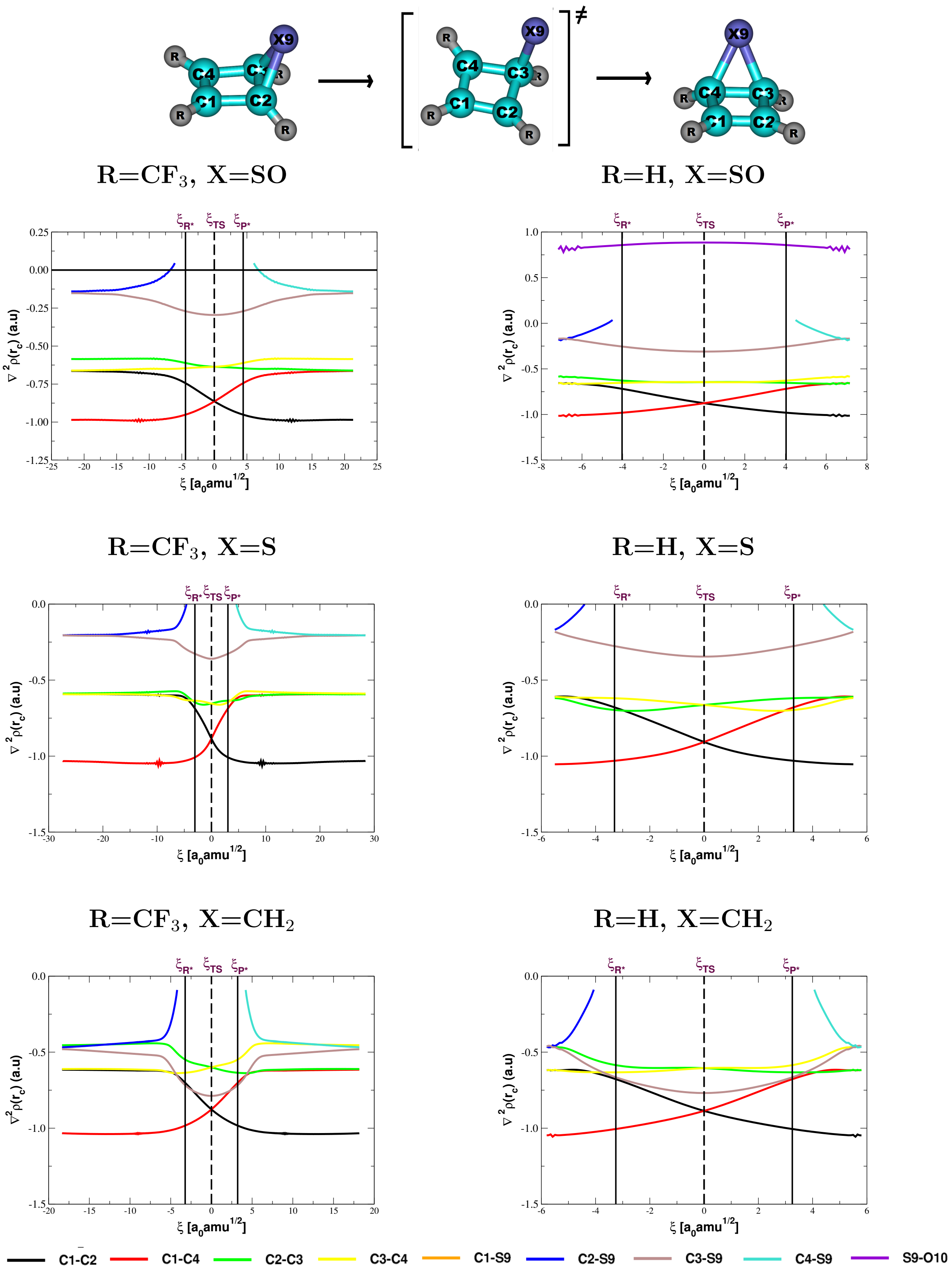

Figure S5: Laplacian of the electron densities in bond critical points along the IRC for one particular step of the Dewar rearrangement.

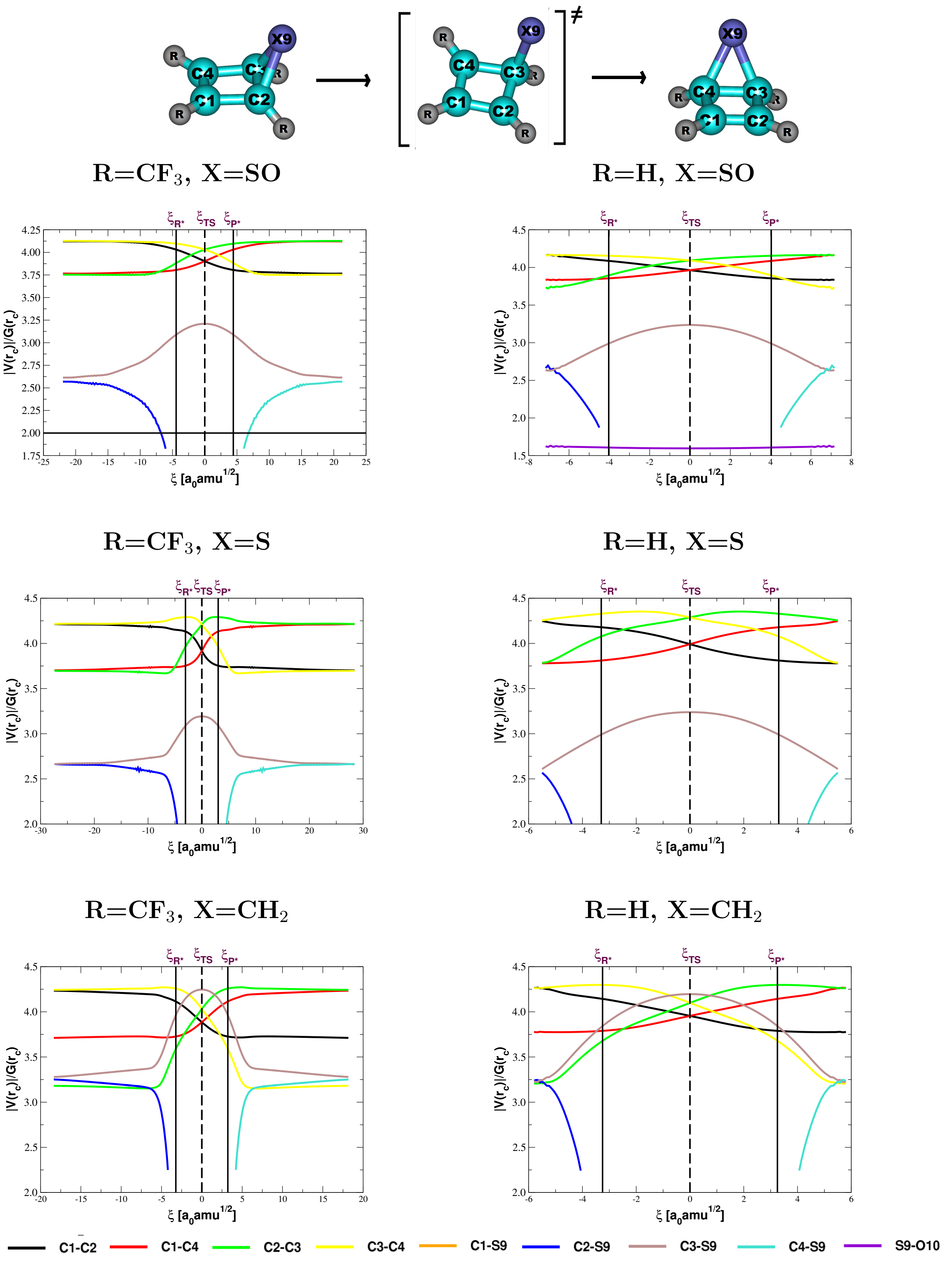

Figure S6: Espinosa's criterion in bond critical points along the IRC for one particular step of the Dewar rearrangement.

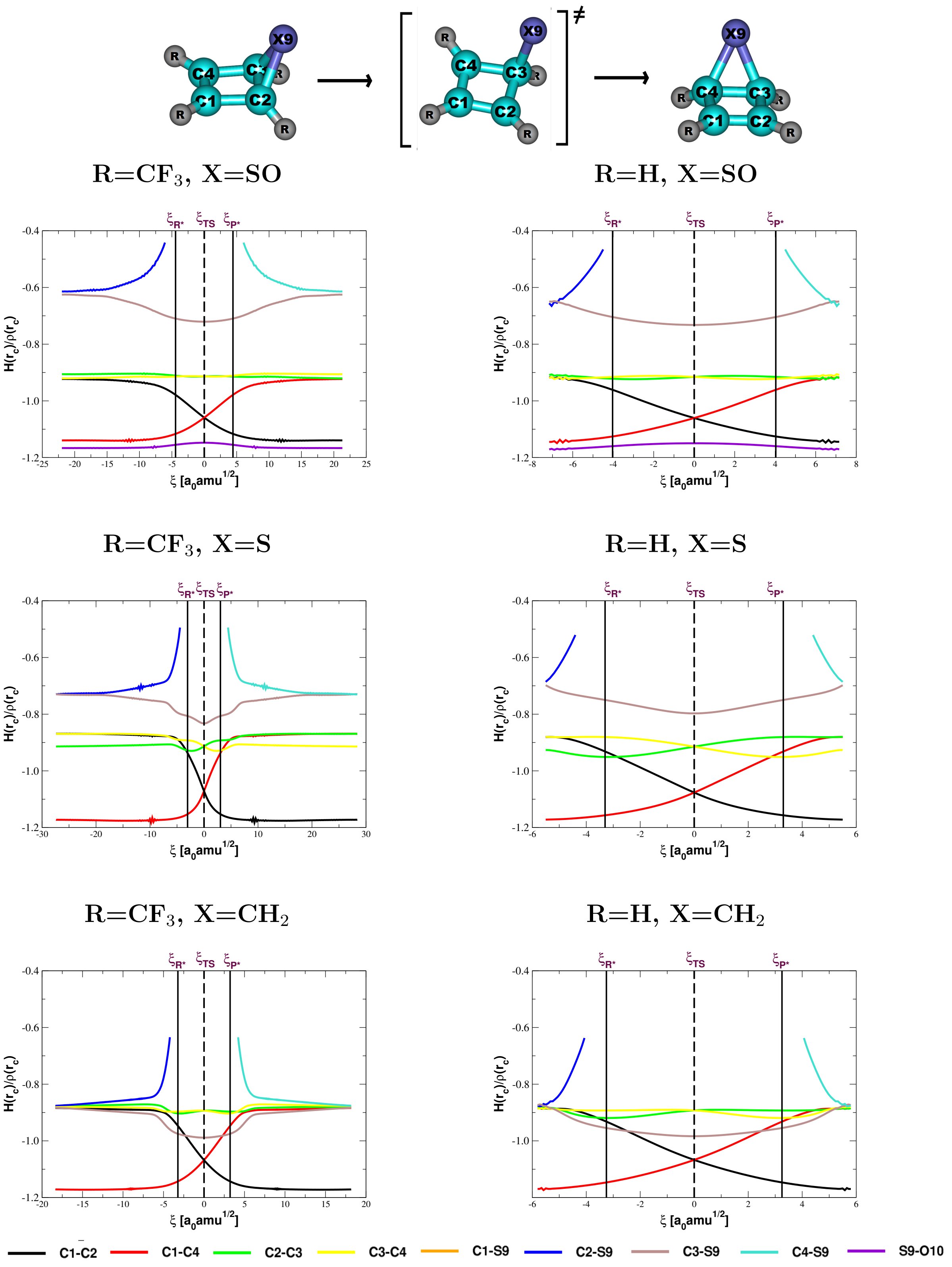

Figure S7: Bond parameter criterion in bond critical points along the IRC for one particular step of the Dewar rearrangement.

2.3 AdNDP Results for all TSs

Table S2: AdNDP derived orbital occupancies of nc-2e bonds in the transition states for all Dewar rearrangements studied in this work. There are marginal contributions from C3 for the R=–CH_2.

Atoms			ON
TS X=–SO R=–H			
C1	C2		1.9859
C1	C4		1.9859
C3	C4		1.9752
C2	C3		1.9752
C3	S9		1.9471
C1	C2	C4	1.9121
C2	C4	S9	1.7340
TS X=–SO R=–CF_3			
C1	C2		1.9762
C1	C4		1.9762
C3	C4		1.9598
C2	C3		1.9598
C3	S9		1.9394
C1	C2	C4	1.8878
C2	C4	S9	1.7144
TS X=–S R=-H			
C1	C2		1.9865
C1	C4		1.9865
C3	C4		1.9753
C2	C3		1.9753
C3	S9		1.9585
C1	C2	C4	1.9611
C2	C4	S9	1.9744
TS X=–S R=–CF_3			
C1	C2		1.9750
C1	C4		1.9750
C3	C4		1.9608
C2	C3		1.9608
C3	S9		1.9496
C1	C2	C4	1.9310
C2	C4	S9	1.9356

TS X=–CH_2 R=–H				
C1	C2			1.9871
C1	C4			1.9872
C3	C4			1.9635
C2	C3			1.9636
C3	C9			1.9709
C1	C2	C4		1.9527
C2	C3	C4	C9	1.9984

TS X=–CH_2 R=–CF_3				
C1	C2			1.9758
C1	C4			1.9758
C3	C4			1.9454
C2	C3			1.9453
C3	C9			1.9653
C1	C2	C4		1.9207
C2	C3	C4	C9	1.9391

3 Optimized geometries at the PBE0-D3/def2-TZVP level

3.1 Cartesian coordinates for all transition states

TS X=–SO R=–H; E= -627.817510104 Hartrees; Imaginary Freq= -157 cm^{-1}

6	0.893821	-1.666878	0.000000
6	0.932541	-0.703988	1.013880
6	0.932541	0.391362	0.000000
6	0.932541	-0.703988	-1.013880
1	0.728831	-2.735998	0.000000
1	0.891981	-0.700708	2.092410
1	1.455741	1.339612	0.000000
1	0.891981	-0.700708	-2.092410
16	-0.864799	0.352742	0.000000
8	-1.535049	1.656862	0.000000

TS X=–SO R=–CF_3; E= -1975.31949047 Hartrees; Imaginary Freq= -122 cm^{-1}

6	-1.119246	0.000059	-0.190535
6	-0.160854	-1.013669	-0.125222
6	0.931656	-0.000039	-0.054297
6	-0.160757	1.013700	-0.125217
16	0.745286	-0.000021	1.747689
8	1.967038	-0.000065	2.525347

6	2.245431	-0.000119	-0.783437
6	-0.162205	-2.504518	-0.138059
6	-0.161966	2.504548	-0.138070
6	-2.617720	0.000115	-0.118521
9	2.954929	1.079448	-0.472403
9	2.954837	-1.079718	-0.472302
9	2.038946	-0.000171	-2.100716
9	0.926046	-2.982506	0.466157
9	-0.167473	-2.965914	-1.392273
9	-1.236716	-2.994580	0.477362
9	-3.111854	-1.079110	-0.720104
9	-3.111779	1.079420	-0.720021
9	-3.039300	0.000080	1.146925
9	-0.167093	2.965929	-1.392292
9	-1.236480	2.994719	0.477259
9	0.926280	2.982447	0.466222

TS X=–S R=-H; E= -552.627223213 Hartrees; Imaginary Freq= -379 cm^{-1}

6	0.625963	-1.543349	0.000000
6	0.615955	-0.567701	0.994552
6	0.615955	0.553153	0.000000
6	0.615955	-0.567701	-0.994552
1	0.468214	-2.612398	0.000000
1	0.492234	-0.560440	2.069677
1	1.357068	1.348275	0.000000
1	0.492234	-0.560440	-2.069677
16	-1.103295	0.946162	0.000000

TS X=–S R=–CF_3; E= -1900.12742585 Hartrees; Imaginary Freq= -391 cm^{-1}

6	0.000093	1.007090	-0.219329
6	-0.995448	0.059357	0.008916
6	-0.000131	-1.038356	0.217553
6	0.995415	0.059142	0.008917
16	-0.000173	-1.013528	1.971578
6	-0.000220	-2.315243	-0.593891
6	2.494550	0.097210	0.072884
6	0.000302	2.500121	-0.276015
6	-2.494577	0.097745	0.072855
9	1.078579	-3.043504	-0.331435
9	-1.079283	-3.043212	-0.331697
9	-0.000024	-2.024861	-1.898814
9	-2.990096	-1.007573	0.611676
9	-2.967922	0.206686	-1.172255
9	-2.926286	1.144929	0.768864
9	2.967964	0.206083	-1.172204
9	2.989814	-1.008229	0.611699
9	2.926445	1.144290	0.768932

9	0.000439	3.033919	0.946636
9	-1.081194	2.941129	-0.917130
9	1.081884	2.940793	-0.917227

TS X=–CH_2 R=–H; E= -193.807827198 Hartrees; Imaginary Freq=-357 cm^{-1}

6	1.302045	0.000019	-0.285904
6	0.461976	-1.012375	0.176419
6	-0.554510	-0.000019	0.642448
6	0.461938	1.012379	0.176434
1	2.190618	0.000041	-0.905074
1	0.382906	-2.079996	0.027504
1	-0.992359	-0.000036	1.636133
1	0.382835	2.080001	0.027545
6	-1.420882	-0.000006	-0.515219
1	-0.959658	-0.000007	-1.490376
1	-2.507746	0.000015	-0.460806

TS X=–CH_2 R=–CF_3; E= -1541.32295481 Hartrees; Imaginary Freq= -384 cm^{-1}

6	-0.000199	0.944335	-0.179626
6	-1.013655	0.012770	0.019820
6	0.000228	-1.064086	0.300635
6	1.013669	0.013138	0.019583
6	0.000429	-0.988198	1.741399
1	0.000261	-0.009193	2.194506
1	0.000734	-1.864304	2.384494
6	2.493449	0.069559	0.170954
6	-0.000486	2.436972	-0.285479
6	-2.493467	0.068645	0.171035
6	0.000421	-2.396322	-0.402844
9	-1.077177	-3.107550	-0.066208
9	1.078070	-3.107368	-0.065976
9	0.000548	-2.238351	-1.722279
9	2.962377	-1.036214	0.749311
9	2.887677	1.118482	0.902693
9	3.078238	0.186046	-1.026479
9	1.080520	2.865607	-0.934118
9	-0.001877	2.999470	0.928059
9	-1.080382	2.865059	-0.936330
9	-2.961944	-1.036457	0.751027
9	-3.078276	0.183042	-1.026602
9	-2.888143	1.118524	0.901141

3.2 Cartesian coordinates for all minima

Equilibrium Geometry X=–SO R=–H; E= -627.830718219 Hartrees			
6	1.689268	-0.676270	-0.209469
6	0.464754	-0.749818	0.632549
6	0.465557	0.751898	0.630929
6	1.689562	0.675353	-0.210973
1	2.227833	-1.426327	-0.773609
1	0.140578	-1.431501	1.407022
1	0.139929	1.435395	1.403144
1	2.229014	1.424149	-0.776040
16	-0.827952	-0.000775	-0.461934
8	-2.168121	0.000463	0.134026

Equilibrium Geometry X=–SO R=–CF_3; E= -1975.33109033 Hartrees			
6	0.881096	0.697649	-0.113917
6	-0.590698	0.733974	0.075859
6	-0.553660	-0.766042	0.073162
6	0.916778	-0.653269	-0.110616
16	-0.759492	-0.031513	1.790769
8	-2.101276	-0.069905	2.334664
6	-1.491258	-1.766015	-0.531915
6	-1.582913	1.695694	-0.502763
6	1.953791	-1.731824	-0.042821
6	1.861739	1.828663	-0.061772
9	-1.168490	-2.995517	-0.128385
9	-2.750379	-1.527526	-0.193191
9	-1.402287	-1.736960	-1.864328
9	-2.826888	1.369474	-0.180747
9	-1.486600	1.719318	-1.834341
9	-1.337885	2.927586	-0.053297
9	1.573652	2.720048	-1.012085
9	3.110925	1.424191	-0.238157
9	1.780276	2.452517	1.117751
9	1.889448	-2.497946	-1.133579
9	3.184875	-1.250806	0.054246
9	1.721447	-2.512104	1.017122

Equilibrium Geometry X=–S R=–H; E= -552.662508078 Hartrees			
6	-1.362680	0.667369	-0.247146
6	-0.082125	0.747256	0.559949
6	-0.080279	-0.745240	0.561405
6	-1.361903	-0.668856	-0.245981
1	-1.956249	1.426329	-0.739227
1	0.125116	1.378475	1.414506
1	0.122376	-1.373768	1.419178
1	-1.953474	-1.429436	-0.737927
16	1.311509	-0.000298	-0.320368

Equilibrium Geometry X=–S R=–CF_3; E= -1900.16967336 Hartrees

6	0.688938	-0.802882	0.019690
6	0.729497	0.673128	0.393133
6	-0.767367	0.630879	0.391256
6	-0.644119	-0.840551	0.018576
16	-0.029463	0.966429	1.993919
6	-1.730066	1.574453	-0.283338
6	-1.727062	-1.865790	-0.076577
6	1.829138	-1.764564	-0.076001
6	1.639354	1.667701	-0.282270
9	-2.976732	1.289782	0.099434
9	-1.488855	2.845424	0.001441
9	-1.663317	1.423422	-1.608480
9	1.332795	2.924183	0.003521
9	1.577824	1.513601	-1.607603
9	2.900219	1.447881	0.097334
9	-2.560906	-1.563888	-1.074616
9	-2.434812	-1.869893	1.057575
9	-1.259913	-3.088696	-0.282880
9	2.549445	-1.713244	1.049442
9	2.632553	-1.426187	-1.087466
9	1.431870	-3.015397	-0.262091

Equilibrium Geometry X=–CH_2 R=–H; E= -193.863695214 Hartrees

6	-1.077645	-0.668329	0.095948
6	0.345961	-0.756270	-0.403808
6	0.346438	0.756112	-0.403655
6	-1.077509	0.668625	0.095521
1	-1.760069	-1.418815	0.477071
1	0.733648	-1.393602	-1.187080
1	0.734716	1.393183	-1.186728
1	-1.759487	1.419494	0.476730
6	1.249032	-0.000243	0.538229
1	1.026626	0.000241	1.600678
1	2.306902	0.000127	0.285924

Equilibrium Geometry X=–CH_2 R=–CF_3; E= -1541.38464175 Hartrees

6	-0.666886	-0.778779	-0.051500
6	-0.755620	0.672256	-0.471842
6	0.754589	0.673327	-0.471836
6	0.667919	-0.777840	-0.051519
6	-0.000645	0.863649	-1.760748
1	-0.000083	0.067785	-2.495387
1	-0.001346	1.876352	-2.149780
6	1.775200	-1.776368	0.043310
6	-1.772607	-1.779025	0.043375
6	-1.696929	1.675652	0.113090

6	1.694454	1.678047	0.113146
9	1.423349	2.913816	-0.309127
9	2.952057	1.393724	-0.242240
9	1.641936	1.675754	1.446167
9	2.463782	-1.790544	-1.105806
9	1.345094	-3.007075	0.284414
9	2.623724	-1.434870	1.016180
9	-1.340600	-3.008781	0.285970
9	-2.460291	-1.795440	-1.106243
9	-2.622439	-1.438021	1.015275
9	-1.427147	2.911885	-0.308711
9	-1.644908	1.673010	1.446124
9	-2.954050	1.389915	-0.242856
